# How do we identify potential perpetrators of indirect bullying and how do we help them? A review of the characteristics that are associated with perpetration and can be targeted through prevention and intervention

**DOI:** 10.3389/fpsyg.2025.1595958

**Published:** 2026-01-07

**Authors:** Laura M. Crothers, Jered B. Kolbert, Ara J. Schmitt, Jessica Cowley, Kayla Perfetto, Athena Vafiadis, Alexandra Zawodny

**Affiliations:** Department of Counselor Education and School Psychology, Duquesne University, Pittsburgh, PA, United States

**Keywords:** indirect bullying, intervention, perpetrators, relational aggression, Tier II and Tier III intervention

## Abstract

Identifying those who are likely to become perpetrators of bullying may help direct prevention strategies to those who are most at risk of engaging in peer victimization. Meta-analyses have suggested that antibullying interventions are modestly effective only for students in grades 7 or below. Such interventions are typically Tier I programs of a multi-tiered system of support provided to all students. Some research has examined the cognitive and psychosocial predictors of a type of bullying, relational and social aggression, most often used by those in middle school or above, with limited ability of various cognitive profiles to predict perpetration behaviors. Therefore, this article explores which intrapersonal factors, both cognitive and psychosocial, signify increased susceptibility to perpetrating relational and social aggression from middle childhood through adolescence, as a means of developing targeted preventive strategies for this population. A review of existing interventions for indirect bullying is provided, and recommendations are made for additional strategies for addressing these behaviors through Tier II and III efforts.

## Introduction

Bullying in the United States of America (US) is the most common form of school violence and is a significant academic, emotional, behavioral, and health issue (Thomas et al., 2015). As of 2023, nearly 20% of high school students in the US reported being bullied at school or on school grounds (Centers for Disease Control and Prevention [CDA], 2024a). Data from the Youth Risk and Behavior Survey Data Summary and Trends Report: 2013–2023 and 2021–22 School Crime Supplement (National Center for Education Statistics and Bureau of Justice Statistics) suggest that the prevalence of bullying is 26.3% in middle school and 15.7% in high school, and higher among female students (21.8%) than among male students (16.7%; Centers for Disease Control and Prevention [CDA], 2024b).

Rates of bullying tend to be consistent and high for minoritized students, including biracial students, LGBTQ+ students, and students with disabilities, compared to their White, straight, non-disabled peers (Centers for Disease Control and Prevention [CDA], 2024a; Hong et al., 2020; Wells et al., 2019). At school, children with more externalizing and behavioral problems, as well as students who come from poverty and unsafe neighborhoods, tend to be bullied by their peers at higher rates (Iyanda, 2022). Research by Joo et al. (2023) found that among a sample of US adolescents, 42% reported that peers bullied them due to biases related to their multiple social identities, which contributed to an increased fear of school and school avoidance.

Despite the frequency of bullying among those in minoritized groups and in non-Western nations, most research has focused on White children and adolescents in the US and Europe. In particular, studies on indirect bullying (the focus of this article) are even less encompassing of various ages, racial and ethnic groups, linguistic groups, cultures, and nations. Therefore, it is recommended that readers note the underrepresentation of diverse groups of students and ecologies and critically evaluate both the information about the perpetration of indirect bullying and the results of studies designed to assess the effectiveness of interventions for those individuals. It is strongly recommended that future research expand understanding of cultural/contextual differences and broaden studies to include non-Western nations.

### Three essential elements of bullying behavior

The first element of bullying, intentional aggression, is a fairly broad term that implies instrumental aggression is expressed through social, verbal, physical, or electronic (i.e., cyberbullying) behaviors (Cornell and Limber, 2015; Gladden et al., 2014). Cyberbullying includes milder forms of aggression, such as teasing and name-calling, but can also intersect with illegal activities, such as assault or hate crimes (Cornell and Limber, 2015). The second element, an imbalance of power between the perpetrator(s) and victim(s), is the main component that helps differentiate bullying from other types of aggression among peers. Some power imbalances are easy to identify, such as size differences; however, other forms, such as higher peer status, may not be as readily recognizable (Cornell and Cole, 2011; Cornell and Limber, 2015; Olweus, 2013). The third element, repetitive aggressive behavior, tends to be more noticeable and helps differentiate bullying from less severe forms of behavior. However, most acknowledge that a single occurrence of aggression that is severely detrimental to the victim or highly likely to be repeated can be recognized as bullying (Cornell and Limber, 2015; Gladden et al., 2014; Olweus, 2013).

### Types of bullying

There are two general categories of bullying: direct bullying, in which the perpetrator overtly uses aggression in physical and verbal forms, and indirect bullying, in which the perpetrator(s) use more sophisticated, covert methods to antagonize their victim(s) discreetly (Björkqvist, 1994; Múzquiz et al., 2023). While direct bullying and its perpetrators tend to be immediately apparent to both observers and victims, indirect bullying tends to be more subtle. It also may be done anonymously, which may leave victims unaware of the bullying or the identity of the perpetrators until after the behavior has occurred (PACER’s National Bullying Prevention Center, 2019).

#### Direct forms of bullying—physical and verbal

Physical bullying occurs when the perpetrator(s) cause harm or damage to the body or possessions of their victim(s). Such behaviors may include hitting, shoving, or spitting at the victim or damage to property, such as anonymous theft and/or destruction of the victim’s possessions (PACER’s National Bullying Prevention Center, 2019). Verbal bullying occurs when the perpetrator(s) use their words to harm their victim (e.g., teasing, name-calling, taunting, threats of harm; Wang et al., 2009).

#### Indirect forms of bullying—relational and social

Those engaging in indirect bullying (or relational aggression) target the victim’s social status or reputation, whereas direct bullying (verbal and physical) focuses on the characteristics of an individual. Indirect bullying includes purposeful exclusion, spreading rumors, and intentionally embarrassing individuals in public settings, among other attacks on the victim’s social standing or reputation (U.S. Department of Health and Human Services, 2023). While relational bullying is often conceptualized as inherently indirect, it too may have more overt, direct applications, such as ignoring a friend or threatening to end a friendship (Archer and Coyne, 2005). In this paper, we will use Crothers et al. (2009) delineation of indirect bullying into two separate, yet distinct constructs: relational aggression and social aggression. Relational aggression includes behaviors executed within a dyad to threaten the loss of friendship. In contrast, social aggression involves manipulating a group of individuals to inflict social harm on a victim. Both of these types of bullying will be further described later in the article.

#### Indirect forms of bullying—cyberbullying

In addition to the traditional forms of bullying detailed above, research has recently turned its attention to the growing prevalence of cyberbullying. In cyberbullying, the perpetrator(s) use technological methods such as cell phones, the internet, social media, and other forms of electronic communication to antagonize their victim (Campbell et al., 2012; Jungert et al., 2021; Topcu and Erdur-Baker, 2012; U.S. Department of Health and Human Services, 2023).

Though cyberbullying resembles traditional bullying in many regards, it is worth noting that due to the permanence of content posted online and the severity of its potential to affect the victim(s) detrimentally, cyberbullying does not necessarily require repetition of behaviors to be counted as such (Brighi et al., 2019; Jungert et al., 2021; Smith et al., 2009). Furthermore, in cyberbullying, the power imbalance between the perpetrators and their victim(s) has the potential to stem from differences in the technologies available to the involved parties (Campbell et al., 2012; Jungert et al., 2021)

### Who are likely to be bullies/victims

It is important to understand the likelihood that a student will perpetrate or become a victim of bullying to inform prevention and intervention targeting students. Bullying includes different roles for students (bullies, victims, bully-victims, and bystanders). Despite conventional wisdom suggesting that bullies are insecure, research has revealed that bullies tend to be popular and socially intelligent. Because bullies may enjoy high social status, educators may fail to recognize their aggressive behavior toward peers (Hymel and Swearer, 2015).

Research has uncovered that males are more likely to use direct forms of bullying, including verbal and physical peer aggression, while females tend to use indirect bullying behaviors (relational and social aggression; Cornell and Limber, 2015). Understanding which students are likely to use which forms of bullying may allow educators to identify the full range of peer victimization better. In this paper, we will examine the cognitive and personality correlates of indirect bullying perpetration, which may enable early identification of vulnerability to perpetration. Specifically, this article was developed to attempt to contribute to the literature on perpetrators of indirect bullying. While many studies focus on the victims of bullying, few focus on the perpetrators of this common type of peer victimization.

### A more complete look at indirect bullying/relational aggression

Indirect bullying is a set of behaviors exhibited by a perpetrator designed to harm a victim’s emotional or psychological health through threats or damage to their interpersonal relationships, social status, or reputation (Bell et al., 2018; Robers et al., 2013). Such peer victimization may occur in person or through technology (e.g., email, social media, texting, and gaming).

#### Gender and indirect bullying

Within childhood studies, females have appeared to be more likely than males to use indirect bullying instead of traditional (direct) bullying, as indirect forms of aggression rely upon developing strong communication skills, understanding the subtle nuances within social relationships, and a well-developed network of relationships (Bell et al., 2018). Other studies found the opposite, however, with no gender differences in indirect bullying apparent until around 10–11 years of age, when girls engaged in increased relational aggression than boys (Smith et al., 2009).

Crapanzano et al. (2010) found that, among a sample of children in middle childhood, boys’ use of relational aggression may reflect different reasons than their perpetration of physical aggression, as their relational aggression patterns related to distinct categories of reactive aggression alone or combined reactive/proactive aggression. In contrast, relationally and physically aggressive behaviors appear to be related to similar causal factors for girls. However, such gender differences continue to be inconsistent in studies and deserve further investigation—including distinguishing between cisgender and non-cisgender males and females—before we reach firm conclusions.

### Understanding different conceptualizations of relational aggression and indirect bullying

There have been different interpretations of the types and intentions of relational aggression. Voulgaridou and Kokkinos (2018) describe four subtypes of the behavior (i.e., proactive direct, proactive indirect, reactive direct, and reactive indirect) that are the result of the intersections of the functions (i.e., *proactive*—aggressive behaviors motivated by a directed goal and *reactive*—driven by anger or by feelings of low peer acceptance) and forms (i.e., *direct*—awareness of perpetrator and *indirect*—perpetrator or the perpetration is initially unknown to the victim). Another perspective is from Crothers et al. (2009), who differentiated these behaviors into two subcategories, relational and social aggression, by distinguishing them through confirmatory factor analysis on a rating scale they developed. In this article, we will use Crothers et al. (2009) conceptualization of indirect bullying. While Voulgaridou and Kokkinos (2018) conceptualization of indirect bullying is undoubtedly more nuanced, the two-factor explanation introduced by Crothers et al. (2009) is straightforward. It captures the full behavior for intervention purposes.

#### Relational aggression

Crothers et al. (2009) contend that in perpetuating relational aggression, the individual’s primary focus is to influence or directly control the person’s behavior within that one-to-one relationship. Relationally aggressive behaviors are psychologically more intimate than social aggression, which relies upon the group context to inflict emotional hurt. The intent to harm another may be expressed through the exploitation of a friendship, sarcasm, speaking to another in a cold or hostile tone, ignoring, staring, eye-rolling, and “mean” facial expressions. The victim’s self-esteem is typically damaged through these kinds of relationally aggressive behavior (Archer and Coyne, 2005; Crain et al., 2005; Remillard and Lamb, 2005; Simmons, 2002).

When a victim is undermined in this way, they often feel emotionally and socially vulnerable and appease the perpetrator to ingratiate themselves to reestablish the security of the friendship. It may be easier to defer to the perpetrator than cause a conflict that risks the sustainability of the relationship. As adolescents become more reliant upon peers for their sense of self, sense of belonging, and emotional and psychological stability, the potential loss of a friendship may be devastating (Crothers et al., 2005).

#### Social aggression

Crothers et al. (2009) hypothesize that social aggression is the intent to manipulate and damage another’s social status or group membership through either covert or overt means. Social aggression requires manipulating a social group as the vehicle of harm, such as gossiping, spreading rumors, and social isolation. It is more sophisticated than relational aggression since it requires knowledge of social dynamics and the ability to subtly influence or orchestrate others’ behavior to achieve desired outcomes.

Social aggression damages children’s social standing, which may be devastating, given that attachment to friends during middle childhood and adolescence predicts positive social, emotional, and behavioral outcomes (Schoeps et al., 2020). Poor attachment to friends may damage children’s social self-perceptions, which may be associated with conduct problems and emotional difficulties (Schoeps et al., 2020). Furthermore, because of the group mentality involved with social aggression (e.g., following a dominant individual’s lead of disliking a victim), escaping or quelling the abusive treatment may be difficult to achieve among one’s peers (Bell et al., 2018; Comstock et al., 2013).

## Cognitive correlates of indirect bullying

Crothers et al. (2024) reviewed the limited literature regarding cognitive correlates of indirect bullying. Unfortunately, it has proven difficult to identify new studies to understand the relationship between these two constructs since the writing of that article. As previously explained in the Crothers et al. (2024) review, researchers use different terms in the study of indirect bullying (e.g., relational aggression, indirect aggression, social aggression), making it challenging to aggregate study results to explain the phenomenon. Additionally, we have seen a recent morphing of the term “indirect bullying” in the literature, with some researchers seemingly using the term to describe bystanders, but not observable perpetrators, of bullying behavior. All this is to say that additional literature is required to understand cognitive links to indirect bullying.

### Social information processing and indirect bullying

As mentioned, most research on the cognitive contributors to indirect bullying has been devoted to the SIP model. In SIP theory, children’s social behavior is a result of the sequential steps of the processing of social information, including (1) the encoding of social cues, (2) the interpretation of social cues, (3) the clarification of goals, (4) response access or construction, (5) response decision, and (6) behavioral enactment (Crick and Dodge, 1994, 1996). Steeves (2023) found that late adolescent perpetrators of relational aggression conveyed no specific SIP response decision (Assertiveness, Passiveness, Overt Aggression, and Relational Aggression), while socially aggressive participants were significantly less likely to endorse response decisions that are more overtly aggressive or relationally aggressive. Steeves (2023) concluded that this finding is also consistent with research indicating that social aggression is more sophisticated than relational aggression, as it requires understanding social dynamics and roles to achieve one’s own goals through manipulation (Crothers et al., 2019).

### Executive functioning

Executive functioning is the higher-order cognitive skills necessary for goal-directed behavior, mediated by the pre-frontal cortex and providing management and direction to lower-order brain functions (Goldstein et al., 2014; O’Toole et al., 2019; Stuss and Levine, 2002). Executive functions may be categorized as hot (involving emotion) or cold (involving cognition; Ward, 2020). Hot EFs include processing information related to reward, emotion, and motivation (Salehinejad et al., 2023). O’Toole et al. (2019) explain that cool executive functions (EF) are cognitive processes, such as inhibition, working memory, and planning, used to solve abstract, emotionally neutral problems (Zelazo and Müller, 2002).

In later childhood, cool EF (e.g., flexible thinking, planning, and goal setting) was associated with relational aggression among 8–12-year-old children (McQuade, 2017). In 9–13-year-old children, relational aggression predicted future relational aggression, with this effect moderated by strong EFs (McQuade, 2017). These children learned that relational aggression effectively exerted control over peers and helped them to gain social status, likely reinforcing their relationally aggressive behavior.

A recent investigation captured our attention because it helped us understand the possible connection between general cognitive ability and executive functioning, as brain-based constructs, and the expression of indirect aggression. Weinreich et al. (2023) conducted a study of elementary school students. They determined that the presence of a learning disorder (a disorder rooted in theoretical dysfunction of cognitive skills) is not directly related to risk for engagement in bullying. However, the relationship between learning disorder and bullying is indirect through the existence of externalized behavior problems.

This finding is consistent with Kishimoto et al. (2023), who also found that externalizing behavior is related to the perpetration of bullying and pointed to executive functioning constructs, like decreased behavioral inhibition (please see our later discussion of personality and behavioral attributes related to relational aggression). Because a lack of behavioral inhibition has been associated with overt bullying across childhood (Jenkins et al., 2018; Kishimoto et al., 2023; Noten et al., 2020), more research is needed to understand the relationship between inhibitory control and indirect aggression. The numerous studies linking EF to indirect bullying suggest that a strong relationship exists; however, the direction, form, and magnitude of these connections appear to be dependent upon the age, cognitive skills, disability status, and social skills of the individuals.

### Theory of mind and indirect aggression

Theory of Mind (ToM), the ability of individuals to recognize and appreciate their own and others’ mental states to use this information to explain and predict behavior (Premack and Woodruff, 1978), has been proposed as a variable that may explain indirect aggression (Sutton et al., 1999). ToM development permits children to be aware of others’ relationships, possibly teaching them how to be the most hurtful to others through gossiping, manipulation, or social exclusion (Gomez-Garibello and Talwar, 2015). Indeed, studies have found positive correlations between all forms of aggression, including indirect aggression, and ToM skills in preadolescents (e.g., Gini, 2006; Kokkinos et al., 2016a).

Therefore, researchers found that indirect aggression was significantly positively associated with moral disengagement and negatively with ToM in a sample of Greek preadolescents. However, although boys with deficient ToM were more likely to disengage morally from their actions and use relational aggression, poor ToM was only directly connected to relational aggression for preadolescent girls. ToM partially mediated the relationship between moral disengagement and relational aggression in girls (Kokkinos et al., 2016a). As with EF, there are undoubtedly linkages between ToM and indirect bullying, but the strength, direction, and mediation of these relationships are dependent upon gender, age, and cognitive sophistication. Additional study is needed to understand the role of ToM in indirect bullying fully.

### Elaborative and deep processing and indirect bullying

Some may speculate that there is a relationship between cognitive maturity and the perpetration of indirect bullying. In an investigation examining the fourth stage of cognitive development in childhood and adolescence (formal operational thought, as described by Piaget, 1967) and the perpetration of indirect bullying, researchers sought to clarify the role of indirect bullying at the highest level of cognitive sophistication during this developmental period.

The investigators examined the relationship between college students’ learning styles (measured by the Inventory of Learning Processes—Revised; Schmeck et al., 1991) and their use of two forms of indirect bullying (social and relational aggression). In this study, two dimensions of reflective processing (deep and elaborative processing) were found to be related to both forms of indirect bullying (Crothers et al., 2014).

Relational aggression and social aggression accounted for 81% of the variance in deep processing (thinking abstractly, reasoning hypothetically, and solving problems systematically), and 87% of the variance in elaborative processing (encoding new information through personal metaphor and vocabulary; Schmeck et al., 1991). These findings were consistent with the view that indirect forms of aggression require higher levels of social intelligence and social skills than direct bullying (e.g., Björkqvist et al., 2000; Crothers et al., 2014).

### General cognitive abilities and indirect aggression

As the research on cognitive maturity and indirect bullying was established by Crothers et al. (2014), subsequent research by this group extended the inquiry to overall cognitive ability and this type of bullying. In particular, the researchers speculated that verbal acuity and working memory would be related to the perpetration of indirect bullying, as these abilities would represent the savvy required for this type of aggression.

Crothers et al. (2019) administered a standardized measure of intellectual functioning, a measure of indirect bullying, and a measure of prosocial skills. Specifically, the researchers sought to learn if performances on a theory-based, individually administered test of cognitive abilities (*Woodcock-Johnson III Normative Update Tests of Cognitive Abilities*; Woodcock et al., 2007) could be used to understand the utilization of self-reported relational aggression, social aggression, overt aggression, and prosocial skills among late adolescent females.

None of the cognitive abilities assessed by this measure explained a notable amount of variance in indirect bullying, whether relational or social aggression. These results suggest that measures of general cognitive abilities—not directly tied to social contexts—are not particularly useful in understanding the expression of indirect aggression.

## Summary of cognitive predictors of indirect bullying/relational aggression

Although this review article focuses on relational aggression, findings from the overt bullying literature may inform our understanding of the association between cognitive abilities and indirect aggression. Higher IQ and executive functioning are associated with a reduced risk of perpetrating direct bullying (see Liu et al., 2017; Verlinden et al., 2014, for example, experimental studies). These research implications from the Crothers et al. (2024) study of cognitive correlates are that the perpetration of indirect aggression is better understood by flexibility in thinking, the ability to focus on particular elements of social behavior, inhibit some responses, and engage in perspective-taking, and other characteristics associated with cool EF, than by overall intellectual functioning.

### Intervention for bullying

By 2017, while all 50 states in the US had enacted antibullying laws for their schools, no federal laws explicitly prohibited school bullying. Standard components among state regulations, laws, and policies include definitions of bullying, characteristics of bullying behaviors, and comprehensive school district policy requirements (Stopbullying.gov, 2023). Unfortunately, in their earlier analysis of more than 40 statewide antibullying laws, Kueny and Zirkel (2012) noted their limited impact, explaining that such laws failed to address cultural variables within schools and to provide accountability for enforcing them. In a study of the effects of the Iowa Safe Schools Law, bullying rates remained unchanged before and after the law’s passage (McGeough, 2022).

In response to an earlier meta-analysis by Ttofi and Farrington (2011), Yeager et al. (2015) argued that between-study analyses of age-related effects on the effectiveness of antibullying programs may be misleading. These researchers described using within-study analyses that compare the duplicate content delivered at different ages, reducing confounding variables and increasing the precision of age-related estimates. Using a three-level meta-analysis of within-study age moderation, these researchers found that although bullying appeared to be prevented mainly below seventh grade, in eighth grade, and thereafter, the effects of antibullying programs sharply declined to an average of 0 (Yeager et al., 2015). Yeager et al. (2015) analyses suggest that, for students in the tenth through twelfth grades, antibullying interventions are generally associated with iatrogenic, harmful effects.

### Why state laws and antibullying programs are not strongly effective

McGeough (2022) examined the ineffectiveness of state laws in curbing student bullying. These include numerous concerns, from limiting the constitutional right to free speech to a lack of training for educators and school staff in how to engage with perpetrators and victims of bullying. In their meta-analysis of antibullying programs, Ttofi and Farrington (2011) suggest that schools’ failure to monitor student behavior carefully, the use of ineffective consequences, and the omission of parents in preventing and responding to bullying curtail the effectiveness of antibullying interventions.

In a qualitative analysis of teachers’ perceptions of the limitations of the effectiveness of antibullying programs, educators cited the *complexities of bullying*, from the behaviors occurring in areas in which educators were not (off school property or through social media) to the variation in the behaviors (manipulative, covert actions seen in relational and social aggression or the switching of modalities to evade adults’ notice). Teachers also called out the *failure to adapt the developmental level of antibullying activities* to students at each grade level. Some suggested that programming for older students should shift to a more active, problem-based approach in which students find solutions (Cunningham et al., 2015).

Educators also described *implementation factors* that limited the impact of antibullying programs, including top-down selection of programming (lacking teachers’ social validity), insufficient time to learn and implement the antibullying lessons, staff attrition, insufficient resources, and failure to ensure long-term maintenance. Most educators in the study reported not being provided with convincing evidence of the program’s effectiveness and not seeing student behavior change (Cunningham et al., 2015).

Teachers reported that other lessons were moved to make time for the antibullying programming, and even then, there was not enough time to fully implement them. When students brought issues to their teachers, there was sometimes no time to help them talk to each other and work through their problems because of other educational responsibilities. Furthermore, some teachers were concerned about waning support from school boards and administrators or a lack of complete buy-in by colleagues, which created an uneven willingness to change and adopt new practices. Similarly, parental support was not uniform, with some parents actively resisting addressing bullying behavior in their children. Some students were unwilling to participate or cooperate with new policies or programming (Cunningham et al., 2015).

In line with the argument that states’ antibullying laws have not been effective, teachers reported that increased monitoring was not pursued as a preventive measure. Disciplinary measures or the tracking of those involved in bully-victim conflicts were not consistently and thoughtfully implemented. Relatedly, teachers reported that schools failed to apply programs consistently across schools and grades or sustain potentially successful programming. When implementing the antibullying program, some teachers modified the lessons, negatively affecting treatment integrity (Cunningham et al., 2015).

### What should be done from a systems perspective?

Some antibullying researchers have suggested analyzing what shared components of schoolwide policies are working most and least effectively (Wampold, 2015). Funding for antibullying efforts and mental health should be a priority in all schools, as children who perpetrate peer victimization or are bullied have higher rates of depression and suicidality than those not involved in bullying. Additionally, actively monitoring the implementation of the policies must be a part of each state’s oversight of its antibullying law.

Those who are particularly vulnerable to being bullied, such as LGBTQ+ students, need to be protected through school climate interventions that promote social acceptance and the equality of academic and mental health initiatives while decreasing an atmosphere of hostility (Greene, 2006; Vreeman and Carroll, 2007). Effective antibullying initiatives that address broader community norms are needed to combat discriminatory practices. While antibullying practices have enjoyed some success in combating overt bullying, relational and social aggression have not been reduced to the same degree (Greene, 2006; McGeough, 2022).

## Recommendation: targeted Tier II and Tier III intervention for existing and prospective perpetrators

In multiple meta-analyses on the effectiveness of whole-school antibullying intervention programs, the most recent research suggests that the positive effects are modest for children in grades 7 and below and are neutral to iatrogenic (harmful) for 8th-grade students and older. Multiple suggestions have been made in the previous pages to improve these antibullying programs. We will speak to our concerns about antibullying programs, in general, and then to the effects that such programs have upon the perpetration of relational and social aggression in children.

### Multi-tiered systems of support

In multi-tiered systems of support (MTSS), antibullying programming is typically a Tier I intervention. As a brief review, the MTSS framework is a school-based intervention that provides the multiple degrees of support needed to address the range of learning, mental health, and emotional-behavioral concerns students may have. Regarding bullying prevention and intervention, MTSS delivery includes universal, selective, and targeted intervention services (Stopbullying.gov, 2021). After briefly explaining the universal prevention of bullying, we will turn our focus to Tiers II and III for students vulnerable to or already perpetrating relational and social aggression (Stopbullying.gov, 2021).

Tier I universal bullying prevention efforts reduce risk and increase the school community’s resilience. Tier I antibullying interventions often include strategies to improve the school’s overall social and emotional climate and to promote positive social behaviors and inclusiveness among the student body. Tier I interventions may include classroom meetings to reinforce positive behavior and also general strategies for responding to bullying (Stopbullying.gov, 2021).

Tier II antibullying strategies in MTSS are often called “selective prevention” and are designed for children who need intervention beyond the general strategies used with the entire school population. Such youth may be at risk for perpetration or victimization. Relevant to our review, for those who are vulnerable to bullying others, recommended selective prevention often incorporates reinforcing consequences for bullying, using teacher/counselor mediation to address interpersonal conflict, and encouraging peers to defend victims to remove the social benefits of bullying to perpetrators (Stopbullying.gov, 2021). We will review several Tier II intervention programs for relational aggression in the subsequent pages of this article.

Tier III antibullying strategies in MTSS are referred to as “indicated interventions” for students whose needs have not been adequately addressed at Tiers I and II. Tier III intervention has a greater intensity and specificity for the student’s specific needs. The review of Tier III interventions for perpetrators of relational and social aggression is surprisingly sparse. The spirit of Tier III interventions is to support mental health concerns, behavior issues, and academic performance by working with administrators, multiple teachers, school resource officers, family members, and other supportive adults in the student’s life (Stopbullying.gov, 2021). However, information from both the extant literature and clinical impressions from teachers suggests that there is a lack of targeted treatment for existing and potential perpetrators of indirect bullying (Cunningham et al., 2015).

## Intrapersonal and familial contributions to perpetration of relational and social aggression

### Temperament, personality, and personality psychology

Tackett et al. (2014) examined dispositional aspects of relational aggression, exploring the contribution of temperament, personality, and personality pathology. Relational aggression was more strongly associated with temperament traits than with personality traits. Specifically, relational aggression was associated with negative emotionality and limited self-regulation, which has a very similar profile to other youth externalizing problems (Tackett et al., 2013). The authors noted, however, that there were some divergent results: three subcomponents of extraversion were linked to relational aggression, but in contrasting ways: shyness and sociability were positively related to relational aggression, whereas positive emotions were negatively related to it. The authors concluded that such apparent contradictory results may suggest heterogeneity of youth who engage in relational aggression. Such variation is consistent with previous research indicating that relational aggression has been associated with, and without, social maladjustment (Crick et al., 2006; Rose et al., 2004).

### Self-esteem

Self-esteem has been hypothesized to be related to the perpetration of relational and social aggression in two ways. One mechanism of this relationship may be that lower self-esteem, which leads a child to ward off feelings of inferiority or to increase their perceptions of power, may lead them to perpetrate relational aggression, which Weidmann et al. (2022) term the *self-valorizing pathway* (Ostrowsky, 2010). Two studies have found that lower self-esteem predicted later perpetration of relational aggression in children (Fanti and Henrich, 2015; Guerra et al., 2011), whereas another (Rose et al., 2017) did not.

Children with higher self-esteem may use relational aggression to protect their egos; Weidmann et al. (2022) describe this as the *threatened egotism pathway* (Baumeister et al., 2000). High self-esteem may also encourage the use of relational aggression as a goal-oriented behavior to obtain peer acceptance and bolster self-worth (Volk et al., 2014), a phenomenon Weidmann et al. (2022) describe as the *instrumental pathway.* Although theoretically sound, longitudinal studies have not found that perpetration of relational aggression predicted lower self-esteem later in childhood (Özdemir and Stattin, 2011; Rose et al., 2017). Still, other research found that girls, not boys, later experienced higher self-esteem after engaging in relationally aggressive behavior (Pollastri et al., 2010; Weidmann et al., 2022).

Weidmann et al. (2022) surveyed a sample of 674 Mexican-origin California youth in the fifth, seventh, ninth, and eleventh grades regarding their global and domain-specific self-esteem and the perpetration and victimization of relational aggression. However, we will restrict our discussion to only relational aggression perpetration. These researchers found that the perpetration of relational aggression is associated with later lower domain-specific opposite-sex relationships’ self-esteem at the between-person level. Participants also demonstrated that lower self-esteem in the domain of honesty-trustworthiness is associated with a later onset of perpetration. The findings of this research suggest that there are relationships between self-esteem (domain-specific) and the perpetration of relational aggression, although in various directions.

### Psychological maltreatment

Another study also contributed to the understanding of the perpetration of indirect bullying by explaining that psychological maltreatment in childhood predicts the use of relational aggression in adolescence. The mechanism that permits adolescents (in this case, Chinese adolescents aged nine to 19) to engage in this behavior without self-blame is moral disengagement, a process by which individuals reduce the cognitive tension they experience when engaging in immoral behaviors (Wang et al., 2017). Therefore, moral disengagement, as the lack of self-regulation (EF), mediated the relationship between psychological maltreatment (as a victim) and indirect bullying (as a perpetrator), with gender moderating this process. Boys who demonstrated higher moral disengagement in this study also engaged in higher levels of relational aggression, rationalizing their use of this behavior (Ding et al., 2022).

### Moral disengagement

In another investigation, indirect aggression was positively associated with moral disengagement among Greek preadolescents, which, in turn, mediated the effects of callous-unemotional traits and the behavioral activation system on relational aggression (Kokkinos et al., 2016b). Gini et al. (2020) conceptualize moral disengagement as cognitive mechanisms that allow the moralization of actions that would otherwise be considered immoral (thereby reducing cognitive dissonance). Additional research (see Bjärehed et al., 2021) suggests that further implications of this relationship are undoubtedly present.

### Psychopathic traits

Psychopathic traits and indirect aggression have conceptual overlap, as the manipulation of others involved in relational aggression also characterizes children who exhibit higher levels of narcissism-grandiosity traits (e.g., Colins et al., 2014). Given the conceptual similarities and the fact that psychopathic traits have some malleability during childhood (Bégin et al., 2021), researchers have explored whether psychopathic traits are a risk factor for relational aggression.

Psychopathy comprises three dimensions: an affective component (callous-unemotional traits, i.e., lack of empathy and guilt), an interpersonal component (narcissism and grandiosity), and behavioral patterns (impulsivity and irresponsibility; Dong et al., 2014). A 5-year longitudinal study of children entering preadolescence revealed that, after controlling for demographic confounding variables and conduct problems, only narcissism predicted being classified as a high or consistent user of indirect aggression. Callous-unemotional and impulsive-irresponsible traits were not predictors of increasing or stable use of indirect aggression over time (Boutin et al., 2023). Narcissism-grandiosity has also been found to be related to indirect aggression among preadolescents in other investigations (Bukowski et al., 2009; Onishi et al., 2012; Reijntjes et al., 2016).

### Family/parental contributions to relational aggression

Studies have shown that genetic inheritance and environmental factors predict the perpetration of relational aggression. In middle childhood, genetic and sibling environmental factors have been shown to predict the use of social aggression in a sample of identical twins raised within the same family (Slawinski et al., 2019). Social learning theory has been used to explain how parenting behavior influences the development of aggression (Bandura, 1973), suggesting that children learn, observe, and imitate aggressive behavior from their parents.

In a meta-analysis, Kawabata et al. (2011) evaluated 48 studies and uncovered that the warmth and emotional responsiveness of positive parenting were inversely related to children’s relationally aggressive behavior. In contrast, uninvolved, harsh, or psychologically controlling parenting predicted an increased use of relational aggression in children (Kawabata et al., 2011). Kawabata et al. (2011) theorized that children who receive positive parenting are less likely to engage in relational aggression as they acquire social competence and emotional regulation within a safe parent-child relationship.

Several meta-analytic investigations have focused on the role of parental psychological control in childhood relational aggression, given its conceptual similarities to relational aggression, as both include emotional and social manipulation. Parental psychological control involves manipulation of the parent-child relationship (e.g., withdrawal of love), criticism, and personal control (e.g., possessiveness and overprotectiveness). A parent who threatens rejection if their child fails to meet their expectations has been likened to a child who threatens to end a friendship if their friend does not comply with their wishes.

Kuppens et al. (2013) meta-analysis of twenty-three studies revealed that parental psychological control is associated with youths’ use of relational aggression. While this relationship was weak, explaining only three percent of the variance in youth relational aggression, it is comparable to the degree to which parenting is linked to other problems in childhood (e.g., McLeod et al., 2007). The relationship between parental psychological control and the use of relational aggression was more substantial for adolescents than for elementary-aged children. Kawabata’s meta-analysis found that paternal psychologically controlling parenting, but not maternal psychologically controlling parenting, was predictive of the perpetration of relational aggression.

### Child/adolescent interventions to reduce relational aggression

Research regarding the efficacy of intervention and prevention programs to reduce relational aggression is not nearly as common as studies of physical bullying. It is reflected in our inability to locate any meta-analyses for prevention or intervention efforts for relational aggression in the literature. The Office of the Assistant Secretary for Planning and Evaluation (Blase and Fixsen, 2013) published core intervention components to identify and operationalize what makes programs effective. These include a clear description of the context of a program, its core components, the active requirements to operationalize the essential elements so that they can be taught, learned, and implemented in typical settings, and a practical strategy for measuring behaviors and practices reflecting the program’s principles, values, active ingredients, and activities. Finally, when outcomes are not achieved, assessing whether treatment fidelity has been established is critical to determining whether the lack of effectiveness is due to inadequate implementation or flaws in the programming (Blase and Fixsen, 2013).

While Leff et al. (2010) review of relational aggression programs identified nine programs with promising results, including several focused on middle childhood, nearly all have not been subject to rigorous program evaluation, which has traditionally been referred to as the use of randomized controlled trials (RCTs). In educational settings, RCTs are extremely difficult to execute. Lee-Easton et al. (2022) refer to hierarchies of evidence that use other designs as credible evidence of an intervention’s merit. These authors also describe an approach called “best available evidence” to evaluate interventions, recognizing that high-quality RCTs or meta-analyses may not be available for an intervention, as is the case for treating perpetration of indirect bullying. Rigorous evidence may include a range of research designs, and Lee-Easton et al. (2022) advocate for “accepted principles of research quality” that encompass numerous perspectives on acceptable research designs.

With these principles in mind, the programs that will now be described include the Friend to Friend program (Leff et al., 2009), Making Choices and MC Plus programs (Fraser et al., 2004; 2005), Second Step (Van Shoiack-Edstrom et al., 2002), Social Aggression Prevention Program (Capella and Weinstein, 2006), and Sisters of Nia (Belgrave et al., 2004). While we will not review all of them, we wanted to provide a representative sample of this important work. Leff et al. (2010) drew several conclusions from their review of relational aggression programs, including that programs should be culturally sensitive and adopt a systemic approach to prevention that focuses on the school and community contexts.

### Friend to friend program (F2F)

In the F2F Program, interventions are designed to reduce relational and physical aggression, improve problem-solving skills, and increase prosocial behaviors for urban Black 3rd–5th-grade girls (Leff et al., 2015). Ecological-developmental-systems theory (Bronfenbrenner and Morris, 2006) and SIP (Crick and Dodge) were incorporated into the F2F Program, reflecting behavior strongly influenced by their interaction within a social environment. The program includes a leadership component in which participants co-facilitate F2F classroom lessons for their peers, following the hypothesis that demonstrating new problem-solving skills could modify the negative reputations of relationally aggressive children and improve their relationships with peers and teachers.

Social learning theory (Bandura, 1973) is also incorporated through behavioral practice and role-play to enhance social cognitive problem-solving. The 20-session curriculum teaches girls problem-solving strategies using detective analogies and various teaching modalities, including videos, cartoons, and role-plays. Leff et al. (2015) found that Black girls who received F2F exhibited decreased relational aggression and increased social problem skills in comparison to similar girls who were randomly assigned to a homework and study skills intervention. These gains were maintained at a 1-year follow-up.

### Making choices: social problem-solving skills for children (MC)

The MC program (Fraser et al., 2005) is a Tier I intervention for third-grade students based on the SIP model (Crick and Dodge, 1994). The MC program comprises approximately twenty classroom lessons in which students learn to identify and regulate intense emotions and use self-talk to distinguish between harmful responses and responses more likely to maintain friendships. Smokowski et al. (2004) found that, compared with a control group, third-grade students who participated in MC demonstrated higher social contact scores and significantly lower overt aggression. Additionally, high-risk children demonstrated greater improvements than low-risk students.

### MC plus program

Fraser et al. (2005) evaluated a version of the MC called MC Plus, which included teacher and family enhancements. These enhancements involved using behavioral management strategies of providing group-level incentives, creating heterogeneous assigned groups, and using the Good Behavior Game (GBG) (Evertson et al., 1983). Caregivers of the MC plus condition received newsletters regarding the MC lessons, were encouraged to engage in a home-based exercise, and were invited to attend five 90-min informational sessions about the MC curriculum (Fraser et al., 2005). Compared to the routine condition, children who received MC and MC Plus had lower social and overt aggression and greater social competence, and students who received the MC Plus intervention exhibited a lower hostile attribution bias and increased cognitive concentration.

### Social Aggression Prevention Program

The Social Aggression Prevention Program (SAPP) is a school-based group intervention for fifth-grade girls that uses discussion, role-playing, modeling, games, and collaboration to enhance knowledge of relational aggression, increase self-awareness and empathy, promote assertive communication, and provide strategies for social problem-solving (Capella and Weinstein, 2006). Compared with a control group, participants demonstrated significant gains in social problem-solving, and teachers observed increases in prosocial behavior among girls who had higher levels of relational aggression prior to the intervention.

### Sisters of Nia

Sisters of Nia is described by the creators (Belgrave et al., 2004) as a cultural intervention that seeks to address relational aggression among Black girls by enhancing cultural values and beliefs. The fifteen-session small-group intervention involves building trust, increasing knowledge of African culture, promoting hair care and body image, exploring how Black persons are portrayed in American media, emphasizing the value of education, and establishing short- and long-term personal goals. Results of the pilot study indicated that Black girls aged 11–13 who received the intervention demonstrated decreases in relational aggression and increases in ethnic identity.

### Self-affirmation manipulation

Armitage and Rowe (2017) described an intervention in which children aged 11–16, drawn from a community sample in England, received a brief reading about the harm caused by relational aggression. The children were subsequently asked to engage in a self-affirmation manipulation by recalling past acts of kindness. These participants demonstrated a small but significant reduction in relational aggression over 1 month, whereas those in the control condition had slight increases in their use of relational aggression. The researchers concluded that the self-affirmation manipulation enabled participants to process information about the harms of relational aggression more effectively, thereby counteracting the tendency to avoid information threatening one’s self-concept.

### Family interventions

Kawabata et al. (2011) concluded that the small effect sizes for the relationship between parenting and relational aggression negate a recommendation for the use of targeted interventions for the parents of children vulnerable to using relational aggression. However, the effect sizes for the link between parenting and relational aggression are substantial enough to justify the use of universal interventions that focus on the home environment. Unfortunately, we could not identify any intervention that used family intervention or parenting education as a standalone treatment for reducing relational aggression. However, some Tier II intervention programs included parents.

## Limitations and future directions

In the final sections of this paper, we will use the term “relational aggression” to refer to all indirect forms of bullying. First, it is important to recognize that most of these programs have not yet been researched enough to be considered evidence-based practice. For this reason, practitioners and researchers should be cautious and conduct additional investigations (particularly regarding the ecology of the targeted group) before confidently recommending any of these programs. Nevertheless, Leff et al. (2010) identified several school-based relational aggression programs that achieved promising results with preadolescent children. Whereas most bullying prevention programs are universal, most of the relational aggression programs identified by Leff et al. (2010) can be characterized as Tier II interventions, with the primary intervention format consisting of small-group sessions, and many targeting students who had been identified as using relational aggression. A common characteristic of the programs identified by Leff et al. (2010) is a foundation in the SIP model, with sessions designed to target specific SIP deficits.

Leff et al. (2010) recommended that relational aggression programs provide perpetrators with healthy opportunities to pursue social recognition, and it may be argued that the structured group format granted relationally aggressive children an alternative way to connect with other children and satisfy a need for social status. It is possible that Tier I bullying prevention programs have not been enormously successful due to their focus on universal interventions and their failure to consider a primary motivation for perpetrators of relational aggression, the desire for social status, which may potentially be more achievable in the group setting of a Tier II intervention.

There are also emerging risk factors that we have not yet found sufficiently documented in the professional literature: specifically, the role of technology in the use of indirect bullying. There is much anecdotal information describing indirect bullying being perpetrated through technology use (e.g., texting, emailing, social media sites). However, preliminary research has distinguished indirect bullying from bullying being perpetrated through technology (i.e., cyberbullying; Lattanzio, 2018). Lattanzio (2018) used exploratory factor analysis to differentiate cyberbullying and indirect bullying in a sample of early college students. Undoubtedly, indirect bullying is perpetrated through technology. However, the boundary between indirect bullying and cyberbullying remains diffuse at this time.

## Discussion

Our review of the research literature on the intrapersonal correlates of relational aggression may be interpreted in light of the success achieved by many Tier II relational aggression prevention programs. Relational aggression may protect one’s self-esteem by demonstrating power. Narcissism-grandiosity is the one psychopathic trait that has consistently been found to predict relational aggression. Interestingly, Armitage and Rowe’s (2017) finding regarding the effectiveness of engaging in self-affirmation immediately following reading psychoeducation about the harmful effects of relational aggression may address the tendency of relationally aggressive children to “tune out” such prosocial messages in order to protect their self-concept. In summary, these findings suggest motivations for relational aggression, such as demonstrating status and preserving self-concept through relational victimization.

### Small group interventions

Small group interventions may offer relationally aggressive children an alternative, more prosocial context for achieving status. The group’s guided/didactic instruction, involving perspective-taking and role-playing of reciprocal interactions, may provide relationally aggressive children with a new, more mature way of relating that they find personally satisfying. Relationally aggressive children may not have experienced relationships involving mutual reciprocity. Indeed, our review of the research literature revealed that parental contributions to children’s use of relational aggression are small but significant. Along with Leff et al. (2010), we recommend that relational aggression programming include consultation to support parents in pursuing a different way of relating to their child.

### Lack of Tier III interventions

We were not able to identify Tier III interventions designed explicitly for relationally aggressive children from the extant literature base. Using the SIP framework (Crick and Dodge, 1994) for relationally aggressive children who exhibit deficits in interpreting social cues, professional helpers can promote children’s encoding skills via facial recognition practice, asking students to hypothesize the feelings of the person exhibiting the facial display, and contemplating why they may be feeling that way. To reduce hostile attribution bias, relationally aggressive children can be encouraged to expand their attention to central stimuli regarding the overall context (e.g., “What were other children doing?,” “What happened next?”). Relationally aggressive children may be encouraged to think flexibly by being asked to generate alternative hypotheses to perceiving a peer as aggressive (e.g., “What do you think might have been going on for them?”)

### Emotion regulation interventions

For deficits in the emotional regulation of perpetrators, professional helpers should affirm the first emotion expressed or implied by the child, which is often anger, but gradually help the child identify and express the more vulnerable feelings that often underlie anger, which include sadness, hurt, or jealousy. Identification of such vulnerable feelings often generates anxiety and requires self-soothing, so the professional helper should assess whether the child spontaneously engages in such self-soothing or whether it must be provided to the child by the helper through empathic responses (e.g., “I know you are upset, that hurts to think you are losing you friend, I want to help you with this.”). Children can be taught the primary principle of emotion theory: each emotion is associated with a desire/need.

### Narcissism interventions

For relationally aggressive children who show little concern for others, the professional helper can appeal to their self-interest by helping them identify the advantages of prosocial responses, while also taking a longer-term approach to helping them internalize others’ feelings and concerns. Such an approach is consistent with Boutin et al. (2023) recommendation that interventions for preadolescents with psychopathic traits should help them to learn to consider the wellbeing of others and care about how others feel, not just learn to identify *what* they feel. Indeed, children who use relational aggression may have sufficient cognitive empathy, meaning they can take the perspective of others but lack emotional empathy, indicating that they are insensitive to the feelings of others (Crothers and Kolbert, 2008).

Despite several of the relational aggression prevention programs described in this paper appearing to have achieved adequate empirical support to conclude that they are effective, future research is needed to establish the mechanisms of change for these programs. The most significant area of need appears to be the development and evaluation of Tier III interventions for indirect bullying perpetrators. In summary, the research literature on the correlates of indirect bullying/relational aggression and intervention efforts may have implications for bullying prevention programs. We argue that more targeted interventions for perpetrators and their caregivers are necessary for effective bullying prevention.
